# The Cuito catchment of the Okavango system: a vascular plant checklist for the Angolan headwaters

**DOI:** 10.3897/phytokeys.113.30439

**Published:** 2018-11-27

**Authors:** David J. Goyder, Nigel Barker, Stoffel P. Bester, Arnold Frisby, Matt Janks, Francisco M.P. Gonçalves

**Affiliations:** 1 Royal Botanic Gardens, Kew, TW9 3AE, UK Royal Botanic Gardens Kew United Kingdom; 2 National Geographic Okavango Wilderness Project, Wild Bird Trust, South Africa National Geographic Okavango Wilderness Project Luanda Angola; 3 Department of Plant and Soil Sciences, University of Pretoria, South Africa University of Pretoria Pretoria South Africa; 4 South African National Biodiversity Institute, Pretoria, South Africa South African National Biodiversity Institute Pretoria South Africa; 5 Unit for Environmental Sciences and Management, North-West University, Potchefstroom, South Africa North-West University Potchefstroom South Africa; 6 GroundTruth, 9 Quarry Road, Hilton 3245, KwaZulu-Natal, South Africa GroundTruth Hilton South Africa; 7 Herbarium of Lubango, ISCED-Huíla, Sarmento Rodrigues s/n, Lubango, Angola Herbarium of Lubango Lubango Angola; 8 University of Hamburg, Institute for Plant Science and Microbiology, Hamburg, Germany University of Hamburg Hamburg Germany

**Keywords:** Angola, Botswana, Cuando Cubango, Moxico, peat deposits, Namibia

## Abstract

This paper aims to provide a baseline for conservation planning by documenting patterns of plant diversity and vegetation in the upper catchment of the Cuito River. 417 species are recorded from this region. Nine of these are species potentially new to science. Ten species are newly recorded from Angola, with an additional species only recorded previously within Angola from the northern enclave of Cabinda. The 108 new provincial records for Moxico clearly indicate the lack of collections from Angola’s largest province. We note the existence of extensive peat deposits in the Cuito river system for the first time and suggest that one of Barbosa’s vegetation types in the area needs to be reassessed.

## Introduction

Internationally famous for its wildlife, the Okavango Delta wetland in northern Botswana was the 1000^th^ World Heritage Site to be designated by UNESCO and is surrounded by desert. The hydrology and ecology of the Delta are dependent entirely on rainfall in the highlands of central Angola, and the flow of water south and east through the Okavango’s two principal tributaries, the Cuito and Cubango rivers. The Cubango system has been studied extensively in recent years (Oldeman et al. 2013), but little attention has been paid to biodiversity or conservation of the Cuito drainage.

Central and eastern Angola is overlain by deep Kalahari sands formed from uplifted and reworked deposits of an ancient palaeo-lake. The upper catchment of the Cuito and Cuanavale rivers falls mostly within Moxico Province where the plateau lies at an altitude of around 1500m, and the rivers have cut down to an elevation of around 1350 m. The landscape receives rainfall of approximately 1250 mm a year in the headwater lakes region, dropping to around 750 mm at the southern limits of the core study area which is marked by the Menongue – Longa – Cuito Cuanavale road in Cuando Cubango Province. The rainy season lasts from November to April and soils are highly leached. In consequence, they support very little agriculture (Diniz 1973).

Barbosa (1970) assigned the vegetation of the region stretching from just east of Camacupa [General Machado] to Luena [Luso] and south to Longa to vegetation type 17A. This he described as dense, high, mixed (Zambesian and Congolian) miombo woodland with “chanas” or geoxylic-rich grasslands. According to Barbosa, these woodlands comprise *Brachystegia* species (*B.spiciformis* Benth. and *B.longifolia* Benth.) and *Julbernardiapaniculata* (Benth.) Troupin, with some *Guibourtia* species, *Cryptosepalum* species and *Marquesia* species. Around Longa, the vegetation transitions into Barbosa’s vegetation type 24, which he describes as a mosaic of savanna, woodland and dry forest with characteristic woody vegetation containing *Brachystegiabakeriana* Hutch. & Burtt Davy and *Burkeaafricana* Hook.

White (1977) drew attention to the high rainfall highly leached Kalahari sand system and its peculiar flora in a seminal paper on the underground forests of Africa, extrapolating from his knowledge of similar habitats in north-west Zambia. But detailed surveys of the flora of this region are lacking.

**Figure 1.**
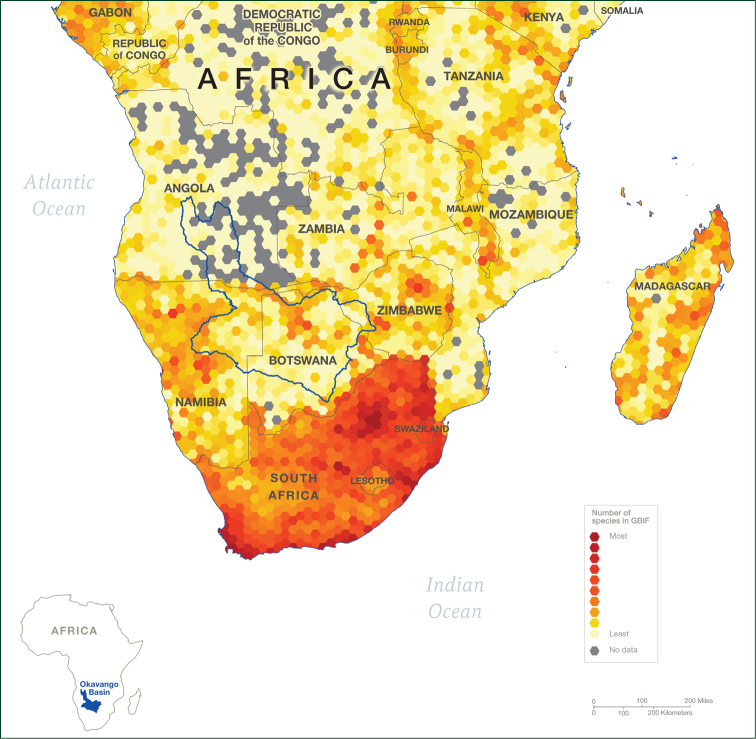
Plant distribution records for southern Africa. Raw data from GBIF (https://www.gbif.org). Note the absence of records for the Upper Cuito River.

Angola is the least intensively inventoried country in southern Africa for plants (Goyder and Gonçalves in press) – this can be seen graphically in the paucity of plant distribution records for the country (Fig. 1) at the start of the National Geographic Okavango Wilderness Project series of expeditions in 2015. Not only is the whole country under-recorded in terms of plants, but the eastern half of the country has very little geo-referenced specimen data (Marshall et al. 2016, Stropp et al. 2016, Sosef et al. 2017). Early collectors such as the Austrian botanist Friedrich Welwitsch collected along the coast, and along routes into the interior as far as Malange Province in the north and the Huíla Plateau in the south, but no further east (Albuquerque 2008, Goyder and Gonçalves in press). Swiss botanist John Gossweiler collected in all of Angola’s provinces over the course of fifty years but spent very little time in central and eastern parts of the country apart from surveys of the Dundo area, Lunda Norte, in 1946 and 1948 funded by the diamond concession DIAMANG (Cavaco 1959, Figueiredo and Smith 2008). In addition, many of Gossweiler’s collections are difficult to localise with outdated place names, and duplicates in herbaria accessible to the authors frequently omit locality data altogether. Slowly, as Angola has become more accessible following the end of the civil conflict in 2002, botanical surveys have resumed in areas of high endemism or conservation concern along the western escarpment (Hind and Goyder 2014, Gonçalves and Goyder 2016, Gonçalves et al. 2016), but the large eastern provinces of Moxico and Cuando Cubango remain poorly documented.

The only major expedition to study parts of the Cuito catchment botanically was the Kunene-Sambesi Expedition led by Pieter van der Kellen, and that covered only the area either side of the present-day Menongue – Longa – Cuito Cuanavale road. The expedition was in the Cuito system from 17^th^ December 1899 to around 4^th^ March 1900, and again between 4–18^th^ April 1900. The botany of the expedition was written up by Warburg (1903) and summarised by Figueiredo et al. (2009) who included notes on the botanist Hugo Baum and on the itinerary. Collections which form the basis of the many species described by predominantly Berlin-based botanists in Warburg (1903) and by subsequent authors were made from the Longa, Cuiriri and Cuito rivers. The area was revisited by Mendes whose 1959–1960 expedition covered the area between Kuvango [Artur de Paiva], Menongue [Serpa Pinto] and Cuito Cuanavale. Prior to the start of the Okavango Wilderness Project many species were known only from this area, and the surveys offered the chance to see if they occurred more widely.

## Material and methods

**Figure 2.**
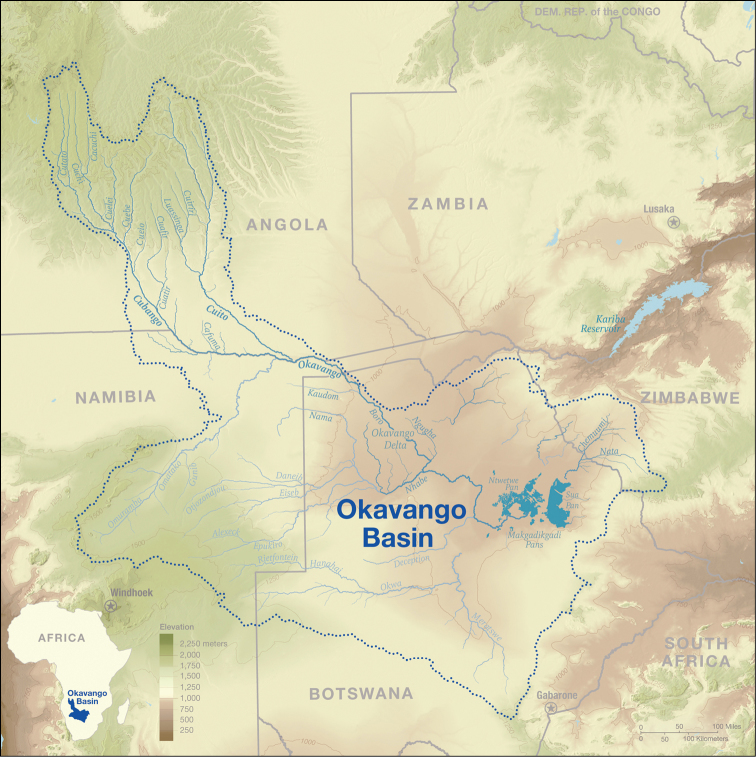
The Okavango Basin and its two principal tributaries the Cuito and Cubango rivers. The core study area is in the more elevated darker green zone of the upper Cuito river.

**Figure 3.**
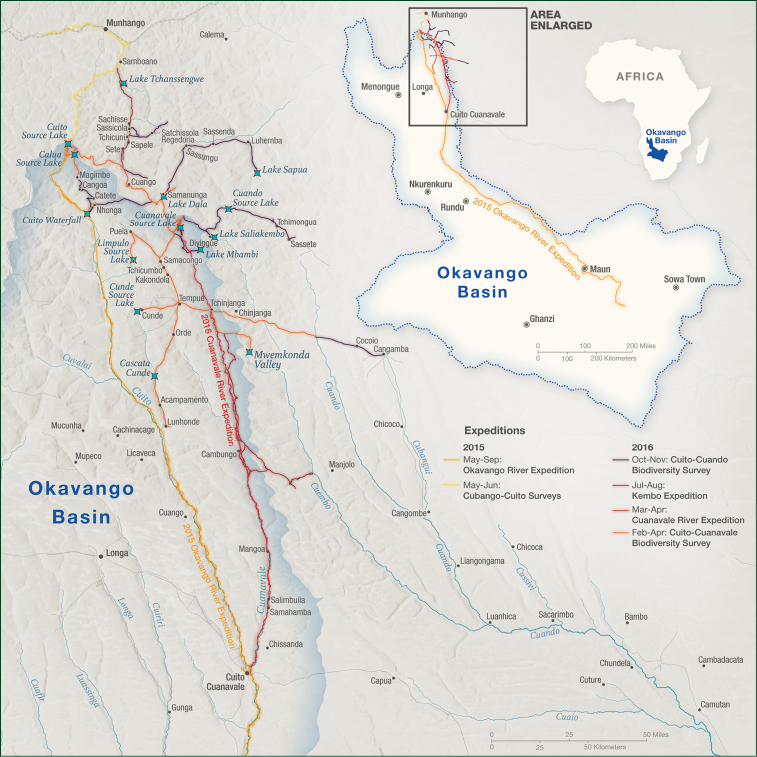
Locations visited during 2015 and 2016 surveys.

**Figure 4.**
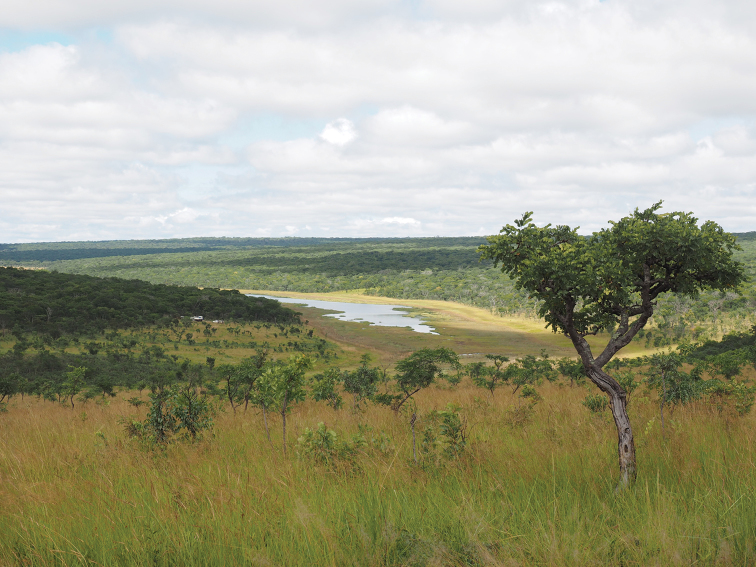
Cuito River source lake, Moxico Province. Extensive moist miombo woodland on the plateau with a few partially cleared areas on the slopes, peaty marsh surrounding the source lake and a narrow strip of fire-maintained grassland between the marsh and the miombo. Photograph D. Goyder.

**Figure 5.**
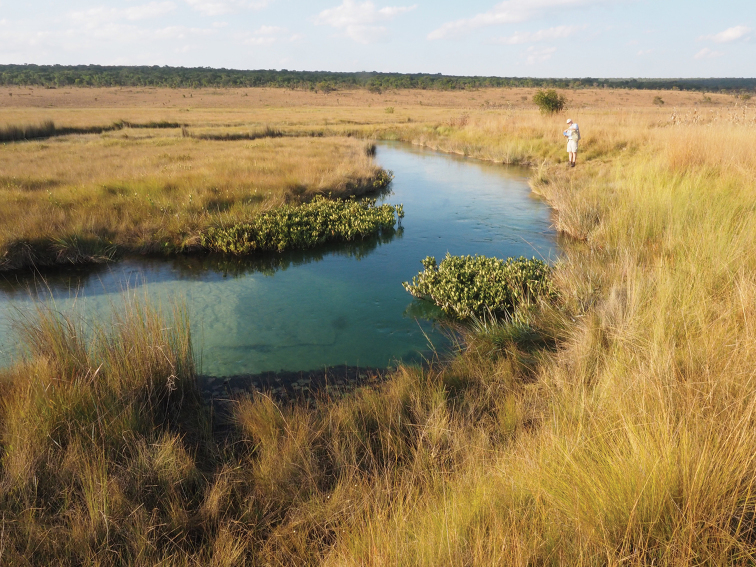
Upper Longa River valley at the southern end of the study area, Cuando Cubango Province. Moist miombo woodland on the plateau with a much broader valley containing more extensive peaty wetlands and fire-maintained grassland zones. The river is fast-flowing in deep sinuous channels with bare sandy bottoms. Photograph D. Goyder.

The core study area is located to the south of Munhango (Figs 2, 3), and fieldwork was centred initially around the source lakes of the Cuito and Cuanavale rivers (Fig. 4), with excursions radiating from these points to the area south of Tempue and to nearby headwater lakes of other river catchments. In addition, more southerly tributaries such as the Longa (Fig. 5), Luassingua and Cuiriri river valleys were accessed from the Menongue – Cuito Cuanavale road. The darker green area towards the top left of Fig. 2 corresponds with the elevated and dissected plateau covered with moist miombo woodland which formed our core study area.

Botanical surveys were conducted at four different seasons to maximise recording of plant diversity – May–June 2015 (dry season), February–March 2016 (height of the rainy season), October–November 2016 (early rainy season) and April 2018 (late rains/early dry season). DG took part in all four surveys and focussed principally on the higher rainfall zones of the catchment between the headwaters and the Menongue – Cuito Cuanavale road (Barbosa vegetation type 17A and its transition to vegetation type 24). FG participated in the third of these surveys, and AF focussed on the Longa and Cuiriri river valleys (transition zone between Barbosa 17A and 24 vegetation types), which were the core of Baum’s study in 1899 and 1900, and which had proved to be of particular botanical interest in earlier surveys. NB, SB and MJ surveyed the Longa area and the catchment south of the Menongue – Cuito Cuanavale road in June 2015.

Plant diversity was mostly assessed through walk-over surveys of each habitat in turn. But for grasses specifically, plots were set up in February-March 2016 following the methodology of Vorontsova et al. (2016) in order to feed into wider continental assessments of natural and anthropogenic grassland diversity. One plot was set up in undisturbed valley grassland near Tempué, a second in grassland possibly cleared from plateau woodland, but apparently long established, above the Cuito source lake, and the third plot was placed in open miombo woodland on the slope immediately adjacent to the Cuito source lake.

The major vegetation types generally form discrete, readily observable units in different parts of the landscape and were categorised informally.

Herbarium collections were made in sets of four where possible and deposited in two Angolan institutions (the National Biodiversity Institute of the Ministério do Ambiente in Luanda and the Lubango Herbarium (LUBA) at ISCED-Huíla), one in the UK (Royal Botanic Gardens, Kew (K)) and one in South Africa (the SANBI Herbarium in Pretoria (PRE)). Plants covered by CITES regulations (*Aloe*, succulent *Euphorbia*, Orchidaceae) were deposited only in Angolan institutions, and identified from photographs. Plants were dried on a frame over a gas burner, using aluminium corrugates to transmit heat and dry air through the press. Collections were identified principally by DG at Kew by reference to the unrivalled tropical African collections and literature held there. Expert opinion was sought from specialists in particular plant groups: Gill Challen – Euphorbiaceae, Phyllanthaceae; Phillip Cribb – Orchidaceae; Iain Darbyshire – Acanthaceae, Linderniaceae, Orobanchaceae; Sebsebe Demissew – *Asparagus*; Peter Goldblatt – *Gladiolus*; Nicholas Hind – Compositae; Isabel Larridon – Cyperaceae; Gwylim Lewis – Leguminosae; Mike Lock – Xyridaceae, Zingiberaceae; Inger Nordal – *Crinum*; Jorge Paiva – *Polygala*; Alan Paton – Lamiaceae; Sylvia Phillips – Eriocaulaceae; Brian Schrire – *Indigofera*; Andre Schuiteman – Orchidaceae; Maria Vorontsova – Gramineae; Kaj Vollesen – Acanthaceae; Martin Xanthos – Cyperaceae, Gramineae.

Angiosperm classification and nomenclature follows APG IV (2016) at family level, and the African Plant Database (version 3.4.0) or the World Checklist of Selected Plant Families (WCSP 2016) in most cases at lower taxonomic levels. Fern and lycopod names follow Roux (2009). On occasion, accepted names diverge from these resources where expert opinion suggests otherwise. Where new country or provincial records are reported, Figueiredo and Smith (2008), recent taxonomic revisions, and searchable online herbarium catalogues (principally Kew (K), the Natural History Museum, London (BM) and the Tropical Institute, Lisbon (LISC)) have been used as the baselines for comparison.

Local usage of plants was documented on 5^th^ and 9^th^ March 2016 thanks to the inhabitants of Samenunga village (12°56'00"S, 018°48'54"E) who explained which plants had medicinal properties, and which were used to make items such as fish traps and beehives. Several cultural artefacts were purchased and deposited in the Economic Botany collections at Kew, where some have since been put on public display. Vouchers of the relevant plants were taken for verification at Kew.

## Results

Approximately 1100 plant collections were made over the course of the four expeditions, with a further 40+ site-based observations recorded.

The principal vegetation types of the core study area are outlined below.

### Vegetation

**Moist miombo woodlands**

Vast swathes of central and eastern Angola are covered in this vegetation. The most common trees we observed were *Brachystegiabakeriana*, *B.longifolia*, *Cryptosepalumexfoliatum*De Wild. subsp.pseudotaxus (Baker f.) P.A.Duvign. & Brenan, *Julbernardiapaniculata*, with frequent *Pterocarpusangolensis* DC., *Erythrophleumafricanum* (Welw. ex Benth.) Harms, BaphiamassaiensisTaub.subsp.obovata(Schinz) Brummittvar.obovata, *Bobgunniamadagascariensis* (Desv.) J.H. Kirkbr. & Wiersema, *Guibourtiacoleosperma* (Benth.) J.Léonard, *Monotesdasyanthus* Gilg., *M.glaber* Sprague, and *Englerophytummagalismontanum* (Sond.) T.D.Penn. Shrubs include *Bauhiniamendoncae* Torre & Hillc., *Bauhiniaurbaniana* Schinz and *Copaiferabaumiana* Harms. Rainfall is generally between 750–1250 mm a year in the upper Cuito catchment. Where the rainfall drops below this, to the south (lower Longa valley and Cuito Cuanavale southwards), other elements such as *Baikiaeaplurijuga* Harms come in, and by M’Pupo Falls, all elements of miombo are replaced by dry thorn-scrub.

Isoberliniaangolensis(Benth.)Hoyle & Brenanvar.lasiocalyx Hoyle & Brenan and *B.spiciformis* are essentially absent from the Cuito catchment, occurring instead on richer substrate to the west. We only noted a single occurrence of *B.spiciformis* in plateau woodland in the Cuito system.

*Brachystegiabakeriana* is most common near the outer margins of Cuito miombo woodland, and where the miombo patches are very small, as in the “fairy forests” near the Cuanavale source, these are dominated by this species. More extensive miombo is on the slopes is usually dominated by *Julbernardiapaniculata*, and some plateau miombo (presumably with different soil composition) by Cryptosepalumexfoliatumsubsp.pseudotaxus, which can form dense, closed canopy stands of miombo forest rather than woodland. Forest lacks the flammable grass layer that is present in woodland and under *Cryptosepalum* we frequently observed the presence of a hummock-forming moss not generally found elsewhere. *Julbernardiapaniculata* was seen as the principle nectar source for honey bees during our 2016 surveys.

**Swamp forest**

We spent a short time in a small patch of swamp forest at the source of the Rio Cuiva (Kwanza drainage). Swamp forest appears to be rare and highly localised in Moxico, unlike in Lunda Norte where extensive formations occur along tributaries of the Kasai River (Congo drainage). The Cuiva swamp forest contained species of Guineo-Congolian affinity such as *Zanthoxylumgilletii* (De Wild.) P.G.Waterman and *Syzygiumowariense* (P.Beauv.) Benth.

**Seasonally burned savannas**

These high rainfall grasslands receive 750–1250 mm of rain a year in the upper Cuito catchment, and are on highly leached Kalahari sand. Eastern Angola contains probably 80% of this habitat, which also extends into parts of NW Zambia and western parts of the DR Congo. This habitat is fire-adapted, and is dominated by grasses or by geoxylic suffrutices, plants with large underground woody biomass and seasonal above-ground shoots. Factors governing whether grasses dominated, or geoxylic suffrutices dominated these areas were not clear. Maurin et al. (2014) argue that across Africa, fire is the evolutionary driver of such lifeforms, whereas Finckh et al. (2016) provide convincing evidence that in upland central and eastern Angola, frost also plays a principal role, with cold air pooling in valley bottoms in the winter dry season and “burning” new shoots. Proximity to the water table limits growth of trees also.

The 2016 surveys took us to several sites with significant expanses of natural or little disturbed grasslands. They were particularly extensive near the confluence of the Cuito and Calua rivers downstream of the Cuito source lake, and the equivalent confluence downstream of the Cuanavale source lake. The third notable site was the Tempué valley grasslands. Grassland diversity plots were placed at three sites – one on the plateau above the Cuito source lake, one in the nearby miombo, and one in the Tempué valley grassland. *Loudetia* species dominated – *L.simplex* (Nees) C.E.Hubb. in open areas and *L.lanata* (Stent & J.M.Rattray) C.E.Hubb. in the woodland. Five to seven grass species were found in each plot. Total grass diversity in the upper Cuito-Cuanavale system was 27 species, the majority (18) occurring in open grassland. Grassland diversity appears significantly higher than in the lower altitude plateau grasslands of Lunda Norte, also dominated by *Loudetiasimplex* (Darbyshire et al. 2011, 2014). *Polygalarobusta* Gürke seems to be associated with diverse natural grassland and could perhaps be considered an indicator of good quality habitat. Another rare species encountered in this environment was the Angolan endemic *Blepharisflava* Vollesen, known from just eight earlier collections. Both of these species are newly recorded from Moxico. A spectacular blue-flowered *Barleria* is new to science and was collected at the Cuito-Calua confluence. Also new to science is a geoxylic species of *Baphia* (Leguminosae), a genus of around 50 species of tree and shrub – the “underground forest” life form had not been recorded in *Baphia* before. This taxon was only seen in one area of the upper Lungué-Bungo catchment, in plains with a rich flora of geoxylic legume species.

*Burkeaafricana* was a common tree in savanna vegetation at the Cuanavale source lake. This was encountered much less frequently in the Cuito source region.

Further south, the upper Longa valley, despite large-scale conversion to rice cultivation, has extensive areas of burned savanna, with some extremely rare species. *Orthantheragossweileri* C.Norman was known only from the type, but we recollected it in the Longa valley in March 2016, and at the Cuanavale source in October 2016 extending its range some 200 km to the north.

**Wetland**

Wetlands tend not to be very diverse botanically, nor to have local endemics. They are however, poorly sampled in Angola.

The extensive peaty wetlands of the Cuito have a much more diverse flora than the rather limited equivalent on the Cubango, which is a much faster flowing river running through a rocky valley. Clump- or tussock-forming plants such as Eriocaulaceae and Xyridaceae are common, while plants such as Droseraceae and Lentibulariaceae are able to supplement the limited nutrients available to other plants by trapping and digesting insects or aquatic invertebrates. Sedges (Cyperaceae) are present but are not as common as preliminary palynological records might suggest (unpublished preliminary results).

The headwater lakes of the Cuito system support a wider range of open water aquatics (true water lilies (Nymphaeaceae) and other aquatics such as *Nymphoides* and *Brasenia*) than is present on the Cubango. One unusual aquatic plant encountered in the fast-flowing upper Longa river was *Mayacabaumii* Gürke (Mayacaceae), a near-endemic and the only old-world representative of this otherwise entirely neotropical family.

Conversely, rocky rapid specialists such as *Hydrostachystriaxialis* Engl. & Gilg (Hydrostachyaceae) and *Inversodicraeawarmingiana* (Gilg) Engl. (Podostemaceae) which are present on suitable portions of the Cubango (Cheek et al. 2017) are completely absent from the Cuito.

Robust river-margin plants include *Gardeniaimperialis* K.Schum. (Rubiaceae) are present throughout both river catchments, while plants such as *Tacazzearosmarinifolia* Oliv. (Apocynaceae) with rheophyic adaptations and requiring a rocky footing are found only on the Cubango.

Many wetland species have their known distributions extended dramatically. *Genliseaangolensis* R.D.Good, for example, was formerly known in Angola from just Cuando Cubango and from one collection in the DR Congo – collections in both 2015 and 2016 demonstrate this species occurs throughout the catchment of the Cuito and Cuanavale rivers (Goyder 2016). Wetland species of *Polygala* and Eriocaulaceae show similar distributions. The photographic record of *Crinumbinghamii* Nordal & Kwembeya from just N of Cuito Cuanavale demonstrates this also, as it was formerly known only from western Zambia (Nordal and Kwembeya 2004, Zimudzi et al. 2008). While extending the known distributions, the new limits reflect the high rainfall, low nutrient Kalahari sand ecology.

The source lakes generally have deep accumulations of unconsolidated peat at their margins. We measured these to a depth of at least five metres at the Cuito source lake. The valleys also have more consolidated peat deposits. Such deposits are rare in tropical Africa. Reiley and Page (2016), in a recent volume on tropical peatland, state that the only significant peat deposits in Angola are on the lower Cuanza River 50 km from Luanda. The upper Cuito and Cuanavale lakes and wetlands seem to have been overlooked, despite reference in the same volume to peaty deposits in the Okavango Delta in Botswana. Analysis of peat cores from these deposits in ongoing at the University of the Witwatersrand in South Africa – pollen trapped in different strata has the potential to shed light on changes in vegetation in the region over thousands of years.

### Plant diversity

417 species of vascular plant were recorded from the core study area of the high-rainfall upper Cuito and Cuanavale drainage system. The Checklist was compiled principally from our own collections from the high-rainfall zone, but with some additional collection made by Hugo Baum in the transition zone to the south. The majority of Baum’s collections from the Cuito drainage system were, however, made in Barbosa’s drier vegetation type 24 even further to the south and are not included in this checklist. Note that Baum’s specimens citing Longa as the locality refer to the river, not to the village currently known as Longa, which is at the southern limit of our core study area, nor to Baixo Longa 100 km to the S, and outside the core study area. A further point of confusion is Warburg’s (1903) map showing the route of the Kunene-Sambesi Expedition places “Hadjon Longa” close to the confluence of the Longa and Cuito rivers even further south in the region of the present-day village of Nankova.

We report nine species from the core study area which are potentially new to science (Table 1). Ten species are newly recorded for Angola with an additional species which had only been recorded within Angola from the northerly enclave of Cabinda. *Orthochilus* is a new generic record for the country (Table 2). But it is the new provincial records that give the clearest indication of how poorly studied the core project area has been to date – we recorded ten new records for Bié Province, ten for Cuando Cubango, and 108 for Moxico – the largest province in Angola.

**Table 1.** Species potentially new to science.

Family	Species	Notes
** Acanthaceae **	*Barleria* sp. nov.	Grassland at the Cuito-Calua confluence. Also in grasslands of upper Lungué-Bungo tributary
** Compositae **	*Vernonia* sp. nov.	Growing in the floating peaty mat at Cuanavale source lake
** Euphorbiaceae **	*Acalypha* sp. not matched	Similar to *A.angustissima* but dioecious. Pyrophytic grassland at head of Rio Cuanavale valley and N of Tempué
** Gramineae **	*Loudetia* sp. nov.	Closest to *L.densispica*. Grassland in Longa river valley
** Lamiaceae **	*Endostemon* sp. nov.	Grassland at the Cuito-Calua confluence, Moxico province
** Leguminosae **	*Baphia* sp. nov.	Found at a single locality in upper Lungué-Bungo catchment
** Linderniaceae **	*Crepidorhopalon* sp. nov.	Open sand in upper Lungué-Bungo catchment
** Orchidaceae **	possibly sp. nov.	Same site as the *Barleria* sp. nov. A eulophioid orchid, but generic affinities uncertain
** Orobanchaceae **	*Buchnera* sp. not matched at Kew	May be undescribed, or a species from DR Congo. Awaiting comment from expert

**Table 2.** Species newly recorded from Angola.

Family	Species	Notes
** Acanthaceae **	*Justiciasubsessilis* Oliv.	Westerly range extension
** Amaryllidaceae **	*Crinumbinghamii* Nordal & Kwembeya	Cuanavale River N of Cuito-Cuanavale. Known also from similar habitats in western Zambia
** Apocynaceae **	*Landolphiacuneifolia* Pichon	Known from NW Zambia and DR Congo
** Apocynaceae **	SecamonedewevreiDe Wild.subsp.elliptica Goyder	Only known previously from western Zambia.
** Cyperaceae **	*Cyperusunioloides* R.Br.	Widely distributed across tropical and subtropical Africa
** Gramineae **	*Schizachyriumclaudopus* (Chiov.) Chiov.	Known from Tanzania, DR Congo and Zambia
** Loranthaceae **	*Englerinagabonensis* (Engl.) Balle	Congolian element, near Cuanavale source. New record for Angola excluding Cabinda
** Orchidaceae **	*Brachycorythiscongoensis* Kraenzl.	Marsh in the Longa and Cuiriri valleys
** Orchidaceae **	*Bulbophyllumjosephi* (Kuntze) Summerh.	Moist miombo woodland in Moxico
** Orchidaceae **	*Orthochilusaurantiacus* (Rolfe) Bytebier	New generic record for Angola
** Rubiaceae **	Gardenia resiniflua Hiern subsp. resiniflua	Suffrutescent form – Longa valley

Botanically, the pyrophytic grassland zone between the marsh and the miombo woodland contains most of the new and interesting species. Over 40 underground forest species (whose nearest relatives are forest trees or shrubs) were recorded from this zone and as part of the ground flora of neighbouring miombo woodlands. They include *Napoleonaeagossweileri* Baker f. (Lecythidaceae), *Trichiliaquadrivalvis* C.DC (Meliaceae), and an undescribed species of *Baphia* (Leguminosae). The *Baphia* was flowering profusely at ground level in the upper Lungué-Bungo catchment, where it occurred in an assemblage of other underground forest species. *Baphia* is a genus of 50 species of trees and shrubs in Africa and Madagascar – this is the first record of a pyrophytic underground forest species in the genus, and it appears to be a species new to science. The diversity of rubber-producing Apocynaceae species in the grassland zone was noted – *Landolphialanceolata* (K.Schum.) Pichon, *L.thollonii* Dewèvre, *Chamaeclitandrahenriquesiana* (Hallier f.) Pichon, and *Raphionacmemichelii* De Wild. were common elements and have been used as sources of natural rubber in earlier times. Other much rarer species of Apocynaceae were also recorded from this zone, including *Orthantheragossweileri* C.Norman, which we found at the source of the Cuanavale river, 200 km north of its earlier known distribution. The new species of *Baphia* will be described separately, along with a more detailed discussion of the geoxylic suffrutex flora of the region.

Thirty-nine legume species were recorded from the upper catchment of the Cuito Cuanavale system and were found in both open and woodland habitats. Most of the miombo trees belong to this family, but there were many herbs also. Other significant elements of the flora include Rubiaceae (26 spp.), Apocynaceae (19 spp.), Lamiaceae (20 spp.) and the genus *Polygala* (Polygalaceae) with 14 species recorded – each habitat had its own suite of *Polygala* species. Monocot diversity was also substantial, with 31 grass species recorded, 17 orchids – mostly in the marsh and grassland zones – and seven species of *Gladiolus* (Iridaceae).

A flame lily species, *Gloriosasessiliflora* Nordal & M.G.Bingham, was recorded from Angola for the second and third times ever, by the headwater team and the Longa/Cuiriri team respectively. It was described from similar marshland habitats in western Zambia in 1998.

## Discussion

Miombo woodland is generally regarded as Zambesian floristically. Nevertheless, we encountered a small but significant element of moist-miombo species with Guineo-Congolian affinities. These include several species of Apocynaceae, *Uvariaangolensis* Welw. ex Oliv. in the Annonaceae, *Paropsiabrazzaeana* Baill. in the Passifloraceae and *Englerinagabonensis* (Engl.) Balle in the Loranthaceae. The small patch of swamp forest at the head of the Río Cuiva is also Guineo-Congolian in affinity with *Syzygiumowariense* (Myrtaceae) and *Zanthoxylumgilletii* (Rutaceae) widely distributed in the Congo Basin and West Africa. Phylogenetically, *Crinumbinghamii* (Amaryllidaceae), a wetland species, is closer to Congolian members of the genus than to Zambesian species (Nordal and Kwembeya 2004).

Cape elements in the flora were restricted to savanna or grassland habitats, sometimes where rocky substrate was encountered locally. *Protea*, *Cliffortia* and *Erica* are three genera with predominantly Cape affinities and species radiations.

Floristic links outside of Africa are demonstrated by a couple of wetland taxa. *Mayacabaumii* (Mayacaceae) is the only African species of an otherwise entirely neotropical genus and family. *Mesanthemumglabrum* Kimpouni (Eriocaulaceae) is allied phylogenetically to a species from Ecuador (Larridon pers. comm., unpublished work in progress). These distributions probably reflect historic transatlantic dispersal events involving birds.

Human population in the region is low, and the few villages we passed are far apart. The low-nutrient landscape does not support much agriculture. Nevertheless, one or two villages grew a diverse range of crops, and neighbouring miombo woodland was cleared for shifting maize and cassava cultivation. Habitat conversion is local but increasing in what is otherwise a remarkably intact ecosystem. Major human impact on the vegetation of the Cuito-Cuanavale system was only really apparent around the town of Cuito Cuanavale, and the section of the Longa valley affected by the large-scale rice project, although many of the grasslands are burned more frequently that they would be without human presence. Also, timber in the upper Lungué-Bungo valley is increasingly targeted as this area is closer to the provincial capital Luena than the rest of the core project area.

Many native plant species are used as medicines or for construction. It is mostly the most common species that are used.

**Figure 6.**
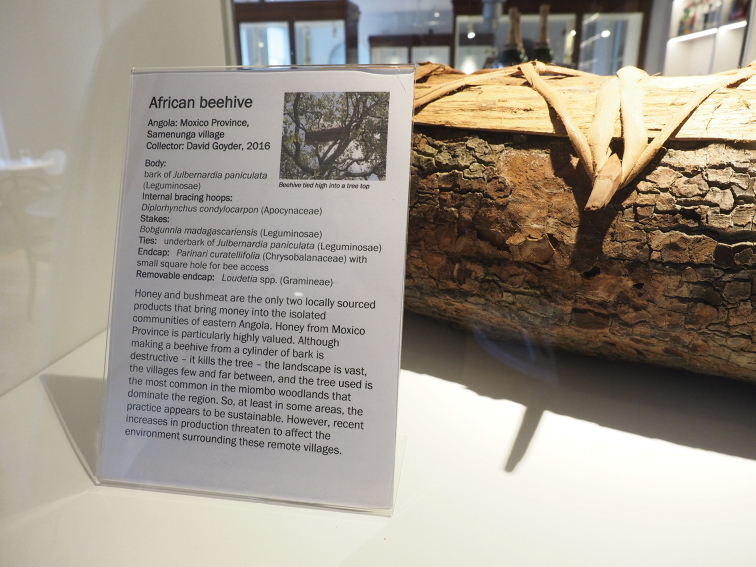
Beehive made in Samenunga village, Moxico Province, now displayed in the Economic Botany collection of the Royal Botanic Gardens, Kew, UK. The body of the beehive is a cylinder of bark from the locally dominant legume tree *Julbernardiapaniculata*. Other species are used to provide stakes, bracing hoops and endcaps. Photograph D. Goyder.

The most frequent miombo tree, *Julbernardiapaniculata*, is not only the principle source of nectar for honey bees but is also the preferred tree for the construction of beehives (Fig. 6). A cylinder of bark is removed from the tree (killing the individual), stapled together with stakes made from another legume timber (*Bobgunniamadagascariensis*), and tied together with stringy underbark from a third (*Julbernardiapaniculata*, *Brachystegiabakeriana* or Cryptosepalumexfoliatumsubsp.pseudotaxus). Internal bracing hoops come from flexible young shoots of either *Diplorhynchuscondylocarpon* (Müll.Arg.) Pichon or Baphiamassaiensissubsp.obovata, the permanent wooden cap at one end is made from *Parinaricuratellifolia* Planch. ex Benth., while the removable cap at the other end is of woodland grasses, mostly *Loudetia* spp. Such traditional methods of construction are destructive but sustainable when population levels are low. However, harvesting of honey and production of beehives is becoming an industry, with some villages boasting of 300+ hives in active use.

Large fish traps were constructed from saplings of *Englerophytummagalismontanum*, tied together with fine bark string as above. The small fishtrap was constructed from the grass *Loudetiadensispica* (Rendle) C.E.Hubb.

**Figure 7.**
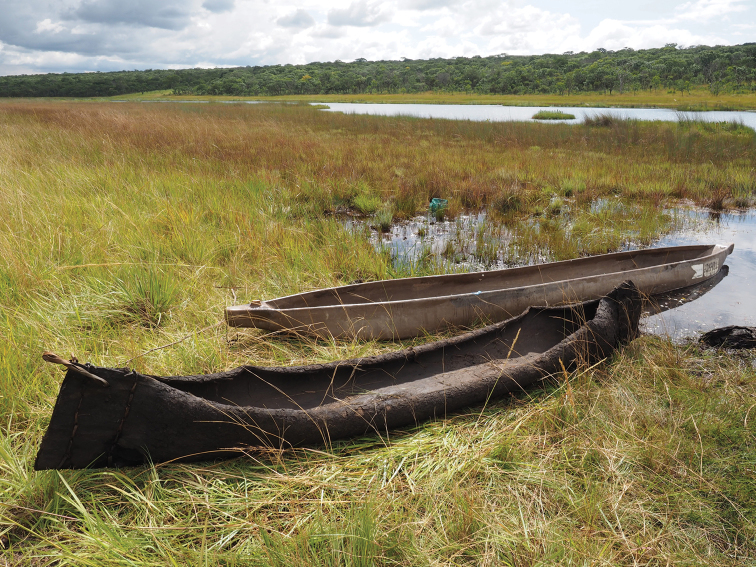
Bark canoe (foreground) made from *Erythrophleumafricanum* (Leguminosae) alongside a fibreglass “dugout” brought in by the National Geographic Okavango Wilderness Project from the Okavango Delta. Cuito source lake. Photograph D. Goyder.

Locally made bark canoes were present in most lakes and major watercourses we visited (Fig. 7). These were generally made from bark of the legume tree *Erythrophleumafricanum* and stitched together as above with strips of stringy underbark from *Brachystegia* or *Cryptosepalum* spp.

It was noted that local people have a detailed understanding of plants with active biological properties in their immediate environment and know how to use these to treat a variety of conditions in the absence of accessible western medicine.

## Conclusions

Over 1100 plant collections were made during the course of the National Geographic Okavango Wilderness Project, the majority from the core project area of the upper Cuito and Cuanavale river catchments. These form the basis of what is undoubtedly the most detailed specimen-based assessment of the vegetation and plant diversity of this region.

The flora of the upper Cuito and Cuanavale system is diverse and endemism is high, although the latter has not been analysed in detail for this study. New records extend the known geographic range of many species 200 km to the north, to the headwaters of the Cuito and Cuanavale rivers. They also underline the need for further surveys in Moxico Province where 108 new provincial records were reported, and provide evidence that the absence of plant records for eastern Angola revealed on the GBIF data map of southern Africa is real, and not a data artefact. All four *Protea* species collected in Moxico had never been recorded there before.

Barbosa (1970)’s vegetation type 17A needs to be critically reconsidered in the light of our findings in this area – we observed a fundamental change in composition of the miombo woodlands east of Cuemba once we moved onto the deep white sands, where several woody species drop out – no *Isoberliniaangolensis* (Benth.) Hoyle & Brenan was seen east of this point, and *Brachystegiaspiciformis* occurred exceptionally rarely. Both *Burkeaafricana* and *Brachystegiabakeriana* are significant elements of the landscape in the headwater lakes region, not just in the transition zone around Longa. We saw no *Marquesia* species in the headwaters zone, but *Monotes* is common. Baphiamassaiensissubsp.obovata, more commonly associated with dry *Baikiaea*-dominated woodland, was a common element of the miombo right up into the headwater region.

We also highlight the existence of extensive peat deposits in the Cuito river system. These are not as extensive as those recently reported from the Congo Basin (Dargie et al. 2017), but must be significant in terms of carbon storage nevertheless.

## Checklist

An annotated checklist of the upper Cuito & Cuanavale drainage system – the flora of high rainfall (annual precipitation more than c. 750 mm), highly leached Kalahari sand deposits from the headwaters to c. 15°S, based prinicipally on 2015, 2016 and 2018 field surveys (Barbosa vegetation type 17A and transition to vegetation type 24).

**Table:** 

**Family**	**Species**	**Habitat**	**Vouchers**	**New Records**
** LYCOPODIOPHYTA **				
** Lycopodiaceae **	*Lycopodiellaaffinis* (Bory) Pic.Serm.	Wetland	Frisby 3027; Goyder 8261	
	*Lycopodiellacernua* (L.) Pic.Serm.	Wetland	sight record 38	
	*Lycopodiellasarcocaulon* (A.Braun & Welw. ex Kuhn) Pic.Serm.	Wetland	Goyder 8298	
** PTERIDOPHYTA **				
** Aspleniaceae **	*Aspleniumaethiopicum* (Burm.f.) Bech.	Humid Forest	Goyder 8329	
** Gleicheniaceae **	*Dicranopterislinearis* (Burm.f.) Underw.	Wetland	Goyder 8396	
**Thelyperidaceae**	*Cyclosorusinterruptus* (Willd.) H.Itô	Wetland	Goyder 8317	Moxico
	*Thelypterisconfluens* (Thunb.) Morton	Wetland	Barker et al. 139	
**ANGIOSPERMAE: MAGNOLIIDS**				
** Annonaceae **	AnnonastenophyllaEngl. & Dielssubsp.nana (Exell) N.Robson	Grassland	Goyder & Maiato 8759; Goyder & Maiato 8843	
	*Artabotrysantunesii* Engl. & Diels	Woodland	Goyder 8436	Moxico
	*Uvariaangolensis* Welw. ex Oliv.	Woodland	Goyder 8034; Goyder 8414; Goyder 8438	
	*Xylopiaodoratissima* Welw. ex Oliv.	Woodland	Frisby 3067; Goyder & Maiato 8806	
	*Xylopiatomentosa* Exell	Woodland	Barker et al. 50; Frisby 3057; Goyder 8027; Goyder 8048; Goyder 8096; Goyder 8288; Goyder 8918	Bié
** Cabombaceae **	*Braseniaschreberi* J.F.Gmel.	Wetland	Goyder 8295	Moxico
** Lauraceae **	Cassytha pondoensis Engl. var. pondoensis	Woodland	Goyder 8104	
** Nymphaeaceae **	*Nymphaeaheudelotii* Planch.	Wetland	Barker et al. 44; Goyder 8259	
	NymphaeanouchaliBurm.f.var.caerulea (Savigny) Verdc.	Wetland	Frisby 4013; Goyder 8296; Goyder 8376	
	*Nymphaeasulphurea* Gilg.	Wetland	Baum 657; Frisby 3050; Frisby 3064; Frisby 3072; Goyder 8097; Goyder 8297; Goyder 8393	
**ANGIOSPERMAE: MONOCOTS**				
** Alismataceae **	*Limnophytonangolense* Buchenau	Wetland	Frisby 3093; Goyder 8375; sight record 15	Moxico
** Amaryllidaceae **	*Boophonedisticha* (L.f.) Herb.	Grassland	Goyder & Maiato 8829	
	*Crinumbinghamii* Nordal & Kwembeya	Wetland	sight record 42	
	*Cryptostephanusdensiflorus* Welw. ex Baker	Woodland	Goyder 8258	Moxico
	*Cyrtanthuswelwitschii* Hiern ex Baker	Wetland	Frisby 4023	Cuando Cubango
** Asparagaceae **	AsparagusafricanusLam.var.puberulus (Baker) Sebsebe	Grassland	Goyder 8439	
	*Chlorophytumcolubrinum* (Baker) Engl.	Grassland	Baum 611	
	*Chlorophytumfasciculatum* (Baker) Kativu	Grassland	Baum 683; Goyder 9495	
	*Chlorophytumsphacelatum* (Baker) Kativu	Grassland	Goyder 9495a	
	*Chlorophytum* sp.	Grassland	Goyder 8263	
	*Dipcadiviride* (L.) Moench	Wetland	Goyder & Maiato 8801	
	*Sansevieriaaubrytiana* Carrière	Woodland	Goyder & Maiato 8838	Moxico
	*Schizocarphusnervosus* (Burch.) Van der Merwe	Grassland	Goyder & Maiato 8779	Moxico
** Asphodelaceae **	*Aloenuttii* Baker	Grassland	Baum 698	
	*Aloezebrina* Baker	Woodland	Goyder 8255	
	*Trachyandraarvensis* (Schinz) Oberm.	Grassland	Frisby 3062; Goyder 8494; Goyder & Maiato 8816; Goyder & Maiato 8820	
** Colchicaceae **	*Gloriosasessiliflora* Nordal & M.G.Bingham	Wetland	Frisby 4035; Goyder & Maiato 8822	Cuando Cubango; Moxico
	*Gloriosasimplex* L.	Woodland	Goyder 8425	Moxico
** Commelinaceae **	*Aneilemaplagiocapsa* K. Schum.	Woodland	Barker et al. 82; Baum 716; Goyder 8244	Moxico
	CommelinaafricanaL.var.lancispatha C.B.Clarke	Woodland	Goyder 8245	
	*Commelinasphaerorrhizoma* Faden & Layton	Woodland	Goyder 8243	Moxico
	*Commelinawelwitschii* C.B.Clarke	Grassland	Baum 814	
	*Cyanotislongifolia* Benth.	Grassland	Goyder & Maiato 8832	Moxico
** Costaceae **	*Costusspectabilis* (Fenzl) K.Schum.	Grassland	Goyder 8947	
** Cyperaceae **	*Abildgaardiaovata* (Burm.f.) Kral	Wetland	Frisby 3041	
	*Bulbostylislaniceps* C.B.Clarke ex T.Durand & Schinz	Grassland	Goyder 8290	Moxico
	*Cyperuschrysocephalus* (K.Schum.) Kük.	Wetland	Frisby 3071	
	*Cyperusdenudatus* L.f.	Wetland	Goyder 8931	
	*Cyperuserinaceus* (Ridl.) Kük.	Grassland	Goyder 8334	
	*Cyperushensii* T.Durand & Schinz	Wetland	Frisby 3081	
	*Cyperuskipasensis* Cherm.	Wetland	Goyder 8939	
	*Cyperusmargaritaceus* Vahl	Grassland	Goyder 8335; Goyder & Maiato 8831; Goyder 8925	
	*Cyperuspectinatus* Vahl	Wetland	Goyder 8294	
	*Cyperusproteus* (Welw.) Bauters	Wetland	Barker et al. 63; Baum 627; Baum 628; Frisby 3009; Goyder 8005; Goyder 8365	
	Cyperusproteus(Welw.)Bautersvar.bellidiflora Welw.	Wetland	Goyder 8936	
	*Cyperusrhynchosporoides* Kuk.	Grassland	Goyder & Maiato 8830	
	*Cyperussubtrigonus* (C.B.Clarke) Kük.	Wetland	Goyder 8940	
	*Cyperusunioloides* R.Br.	Wetland	Goyder 8941	Angola
	*Cyperus* sp. not matched	Grassland	Barker et al. 71; Barker et al. 111	
	Eleocharis acutangula (Roxb.) Schult. subsp. acutangula	Wetland	Goyder 8945	
	Fimbristylis dichotoma (L.) Vahl var. dichotoma	Wetland	Goyder 8942	
	*Fuirenaumbellata* Rottb.	Grassland	Barker et al. 136; Goyder 8924	
	*Lipocarphachinensis* (Osbeck) J.Kern.	Wetland	Goyder 8938	
	*Rhynchosporacandida* (Nees) Boeck.	Wetland	Barker et al. 62; Goyder 8302; Goyder 8368	
	Rhynchosporarugosa(Vahl)Galesubsp.brownii (Roem. & Schult.) T.Koyama	Grassland	Barker et al. 65	
	*Scleriaerythrorrhiza* Ridl.	Wetland	Barker et al. 57; Goyder 8933	
	*Scleriagriegiifolia* (Ridl.) C.B.Clarke	Wetland	Goyder 8239; Goyder 8360; sight record 41	
** Eriocaulaceae **	*Eriocaulonlanatum* H.E.Hess	Wetland	Goyder 8202; Goyder 8369	Moxico
	*Eriocaulonteucszii* Engl. & Ruhland	Wetland	Goyder 8099; Goyder 8364	Moxico
	*Mesanthemumglabrum* Kimpouni	Wetland	Baum 645; Frisby 3065; Goyder 8004; Goyder 8201; Goyder 8238; Goyder 8358	Moxico
	*Mesanthemumreductum* H.E.Hess	Wetland	Barker et al. 115	
	*Syngonanthusangolensis* H.E.Hess	Wetland	Goyder 8237; Goyder 8359	Moxico
	*Syngonanthuswahlbergii* (Wikstr. ex Körn.) Ruhland	Wetland	Goyder 8100	
** Gramineae **	*Aristidanemorivaga* Henrard	Woodland	Barker et al. 108	
	*Brachiariadura* Stapf	Grassland	Barker et al. 59; Goyder 8289	Cuando Cubango
	*Cteniumnewtonii* Hack.	Grassland	Barker et al. 54	Cuando Cubango
	*Digitariamilanjiana* (Rendle) Stapf	Woodland	Goyder 8306	
	Diheteropogon amplectens (Nees) Clayton var. amplectens	Grassland; Woodland	Goyder 8274; Goyder 8285	Moxico
	*Diheteropogonfilifolius* (Nees) Clayton	Grassland	Barker et al. 60; Goyder 8407	Cuando Cubango
	*Eleusinecoracana* (L.) Gaertn.	Wetland	Baum 693	
	*Elymandragrallata* (Stapf) Clayton	Grassland	Barker et al. 98; Barker et al. 105	Cuando Cubango
	*Eragrostisbrainii* (Stent) Launert	Woodland	Goyder 8268; Goyder 8282	
	*Eragrostisthollonii* Franch.	Woodland	Goyder 8284	Moxico
	*Heteropogoncontortus* (L.) P.Beauv.	Grassland	Goyder 8272; Goyder 8404	
	*Hyparrhenianewtonii* (Hack.) Stapf	Grassland	Goyder 8042; Goyder 8923	Bié
	*Leersiahexandra* Sw.	Wetland	Goyder 8930	
	*Loudetiaangolensis* C.E.Hubb.	Wetland	Goyder 8264	Moxico
	*Loudetiadensispica* (Rendle) C.E.Hubb.	Grassland	Barker et al. 109; Goyder 8273; Goyder 8442	Moxico
	*Loudetialanata* (Stent & J.M.Rattray) C.E.Hubb.	Woodland	Goyder 8281	Moxico
	*Loudetiasimplex* (Nees) C.E.Hubb.	Grassland	Goyder 8228; Goyder 8269; Goyder 8403	Bié; Moxico
	*Loudetia* sp. nov. aff. *L.densispica*	Grassland	Barker et al. 55	
	*Miscanthusjunceus* (Stapf) Pilg.	Wetland	Goyder 8299	Moxico
	*Monocymbiumceresiiforme* (Nees) Stapf	Grassland	Goyder 8275; Goyder 8405	Moxico
	*Panicumnatalense* Hochst.	Grassland	Goyder 8271; Goyder 8409	Moxico
	*Pennistetumpolystachion* (L.) Schult.	Ruderal	Goyder 8043	Bié
	*Phragmitesmauritianus* Kunth	Wetland	Goyder 8935	
	*Pogonarthriasquarrosa* (Roem. & Schult.) Pilg.	Grassland	Barker et al. 97	
	*Rhytachnerobusta* Stapf	Woodland	Goyder 8283	
	*Schizachyriumclaudopus* (Chiov.) Chiov.	Grassland	Barker et al. 58	
	*Sporoboluswelwitschii* Rendle	Grassland	Goyder 8291	
	*Trachypogonspicatus* (L.f.) Kuntze	Grassland	Goyder 8913	
	*Tristachyahubbardiana* Conert	Grassland	Goyder 8408	Moxico
	*Tristachyanodiglumis* K.Schum.	Grassland	Barker et al. 72	
	*Tristachyarehmannii* Hack.	Grassland	Goyder 8270; Goyder 8406	
** Hydrocharitaceae **	*Blyxaradicans* Ridl.	Wetland	Baum 827	
	*Otteliamuricata* (C.H.Wright) Dandy	Wetland	Barker et al. 118	
	*Otteliaulvifolia* (Planch.) Walp.	Wetland	Goyder 8929	
** Hypoxidaceae **	*Hypoxiscanaliculata* Baker	Grassland	Goyder & Maiato 8790	
** Iridaceae **	*Ferrariawelwitschii* Baker	Grassland	Frisby 4012; Goyder 8496; Goyder & Maiato 8768	Moxico
	*Gladiolusatropurpureus* Baker	Grassland	Goyder 8498	Cuando Cubango
	*Gladiolusbenguellensis* Baker	Grassland	Baum 632	
	Gladiolus dalenii Van Geel subsp. dalenii	Wetland	Frisby 3029; Goyder 8461	Moxico
	*Gladiolusgregarius* Welw. ex Baker	Woodland	Goyder 8401	
	*Gladiolusgregarius* Welw. ex Baker – anomalous form with filiform leaves and green flowers	Grassland	Goyder 8499	
	*Gladioluslaxiflorus* Baker	Wetland	Frisby 3010; Frisby 3066; Goyder & Maiato 8793	
	*Gladiolusmagnificus* (Harms) Goldblatt	Grassland	Baum 651; Goyder 8497	
	*Gladiolusunguiculatus* Baker	Grassland	Frisby 3025; Frisby 3038; Goyder & Maiato 8777; Goyder & Maiato 8778	
** Mayacaceae **	*Mayacabaumii* Gürke	Wetland	Barker et al. 117; Baum 811	
** Orchidaceae **	*Brachycorythiscongoensis* Kraenzl.	Wetland	Frisby 3068	
	*Bulbophyllumjosephi* (Kuntze) Summerhayes	Woodland	Goyder 8419	
	*Disacaffra* Bolus	Wetland	Goyder & Maiato 8791	
	*Disaochrostachya* Rchb. f.	Wetland	Frisby 4005; Goyder & Maiato 8763; Goyder & Maiato 8796	
	*Disahircicornis* Rchb.f.	Wetland	Frisby 3075	
	*Disawelwitschii* Rchb.f.	Wetland	Frisby 3063	
	*Eulophiaangolensis* (Rchb.f.) Summerh.	Wetland	Frisby 3032	
	*Eulophiahorsfallii* (Bateman) Summerh.	Wetland	Goyder & Maiato 8792	Moxico
	*Eulophialongisepala* Rendle	Grassland	Goyder & Maiato 8753	Moxico
	*Eulophiarolfeana* Kraenzl.	Grassland	Frisby 3095; Goyder & Maiato 8755	Moxico
	*Eulophiaspeciosa* (R. Br. ex Lindl.) Bolus	Grassland	Goyder & Maiato 8774	Moxico
	*Habenariaretinervis* Summerh.	Woodland	Goyder 8220	
	*Orthochilusaurantiacus* (Rolfe) Bytebier	Grassland	Frisby 4002; Goyder & Maiato 8752; Goyder & Maiato 8796	Cuando Cubango
	*Phaiusoccidentalis* Schltr.	Wetland	Goyder & Maiato 8761	Moxico
	*Polystachyaconcreta* (Jacq.) Garay & H.R.Sweet	Woodland	Goyder 8225	
	*Satyriumtrinerve* Lindl.	Wetland	Frisby 3080; Frisby 4001	
	possibly sp. nov.	Grassland	Goyder 8351	
** Smilacaceae **	*Smilaxanceps* Willd.	Ruderal	sight record 16	
** Xyridaceae **	*Xyriscapensis* Thunb.	Wetland	Goyder 8373	
	*Xyriscongensis* Büttner	Wetland	Barker et al. 64; Goyder 8322	
	*Xyrisfoliolata* L.A.Nilsson	Wetland	Barker et al. 128	
	*Xyrisfriesii* Malme	Wetland	Goyder & Maiato 8800	Moxico
	*Xyrisimitatrix* Malme	Wetland	Goyder 8332	
** Zingiberaceae **	*Aframomumalboviolaceum* (Ridl.) K.Schum.	Ruderal	sight record 17	
	*Siphonochilusaethiopicus* (Schweinf.) B.L.Burtt	Grassland Woodland	Frisby 3089; Goyder & Maiato 8769	
	*Siphonochiluspuncticulatus* (Gagnep.) Lock	Grassland Woodland	Frisby 3076; Goyder & Maiato 8770	
**ANGIOSPERMAE: EUDICOTS**				
** Acanthaceae **	Barleria crassa C.B.Clarke subsp. crassa	Woodland	Goyder 8028	
	*Barleria* sp. nov.	Grassland	Goyder 8343; Goyder 8952	
	*Blepharisflava* Vollesen	Grassland	Goyder 8277	Moxico
	*Blepharisglumacea* S.Moore	Grassland	Goyder 8909	
	*Justiciasubsessilis* Oliv.	Grassland	Barker et al. 89	
	*Lepidagathismacrochila* Lindau	Woodland	Baum 779; Goyder 8040; Goyder 8415	Moxico
	*Strobilanthopsislinifolia* (T.Anderson ex C.B.Clarke) Milne-Redh.	Woodland	Barker et al. 107; Goyder 8026	Moxico
	*Thunbergiagossweileri* S.Moore	Woodland	Goyder 8241	Moxico
** Amaranthaceae **	*Mechowiagrandiflora* Schinz	Grassland Woodland	Frisby 4010; Goyder 8112; Goyder 8385	Moxico
** Anacardiaceae **	Lannea gossweileri Exell & Mendonça subsp. gossweileri	Grassland	Goyder & Maiato 8834	
	*Ozoroastenophylla* (Engl. & Gilg) R.Fern. & A.Fern.	Grassland	Baum 662; Frisby 3012; Goyder 8310	Moxico
	*Ozoroaverticillata* (Engl.) R.Fern. & A.Fern.	Grassland	Goyder 8287	Moxico
	*Rhusgracilipes* Exell	Woodland	Goyder 8254	Moxico
	*Rhuskirkii* Oliv.	Grassland	Goyder 8344; Goyder 8911	
** Anisophylleaceae **	*Anisophylleaboehmii* Engl.	Woodland	Goyder 8232	
	*Anisophylleafruticulosa* Engl. & Gilg	Grassland	Barker et al. 46; Baum 808†; Gossweiler 2856; Goyder 8106; Goyder & Maiato 8765	
** Apocynaceae **	*Chamaeclitandrahenriquesiana* (Hallier f.) Pichon	Grassland	Barker et al. 81; Goyder & Maiato 8766; Goyder & Maiato 8807	Moxico
	*Ceropegiaracemosa* N.E.Br.	Woodland	Goyder 8402	Moxico
	*Cryptolepisoblongifolius* (Meisn.) Schltr.	Woodland	Barker et al. 78; Barker et al. 112; Frisby 3037; Goyder 8118; Goyder 8124; Goyder 8300	
	*Diplorhynchuscondylocarpon* (Müll.Arg.) Pichon	Grassland Woodland	Barker et al. 52A; Frisby 3058; Frisby 3061; Goyder 8213; Goyder 8381; Goyder 8445; sight record 1; sight record 8; sight record 36	
	*Glossostelmaceciliae* (N.E.Br.) Goyder	Grassland	Frisby 4033; Goyder & Maiato 8789	
	*Gomphocarpussemiamplectens* K.Schum.	Woodland	Barker et al. 121	
	*Landolphiacamptoloba* (K.Schum.) Pichon	Woodland	Barker et al. 49; Barker et al. 122; Baum 669; Frisby 4004; Goyder 8025; Goyder 8400	
	*Landolphiacuneifolia* Pichon	Woodland	Goyder 8331	
	*Landolphialanceolata* (K.Schum.) Pichon	Grassland	Barker et al. 79; Goyder 8019; Goyder 8266; Goyder & Maiato 8803	
	*Landolphiathollonii* Dewèvre	Grassland	Goyder 8431; Goyder & Maiato 8825 [photographic record]	
	*Orthantheragossweileri* C.Norman	Grassland	Frisby 3051; Goyder 8500; Goyder & Maiato 8827	Moxico
	*Raphionacmeglobosa* K.Schum.	Grassland	Goyder & Maiato 8797	Moxico
	*Raphionacmelinearis* K.Schum.	Wetland	Frisby 3020; Frisby 3035; Frisby 3039; Frisby 3078; Goyder & Maiato 8776; Goyder & Maiato 8856	
	*Raphionacmemichelii* De Wild.	Grassland	Frisby 3026; Goyder & Maiato 8788; Goyder & Maiato 8809; Goyder & Maiato 8771	Moxico
	*Secamonebrevipes* (Benth.) Pichon	Woodland	Goyder 8330	Moxico
	SecamonedewevreiDe Wild.subsp.elliptica Goyder	Woodland	Goyder 8041; Goyder 8223	
	*Strophanthuswelwitschii* (Baill.) K.Schum.	Woodland	Goyder & Maiato 8837	
	*Tabernantheiboga* Baill.	Woodland	Goyder 8226; sight record 18	
	*Xysmalobiumholubii* Scott Elliot	Wetland	Baum 715; Frisby 3034; Goyder & Maiato 8785; Goyder & Maiato 8853	Moxico
** Campanulaceae **	*Lobelia* sp.	Grassland	Barker et al. 116	
	*Wahlenbergiacollomioides* (A.DC.) Thulin	Grassland	Goyder 8906	
	*Wahlenbergia* possibly sp. B of Thulin (1975)	Grassland	Barker et al. 94	
** Caryophyllaceae **	*Polycarpaeacorymbosa* (L.) Lam.	Grassland	Barker et al. 132; Baum 818; Goyder 8457	
** Celastraceae **	*Gymnosporiasenegalensis* (Lam.) Loes.	Wetland	Goyder 8934	
	*Salaciabussei* Loes.	Grassland	Goyder 8292; Goyder & Maiato 8810	Moxico
** Chrysobalanaceae **	*Parinaricapensis* Harv.	Grassland	Barker et al. 130; Goyder 8256	
	*Parinaricuratellifolia* Planch. ex Benth.	Woodland	Goyder 8444	
** Combretaceae **	*Combretumdumetorum* Exell	Woodland	Goyder 8426	Moxico
	*Combretumgossweileri* Exell	Woodland	Goyder 8023	
	*Combretumplatypetalum* Welw. ex M.A.Lawson	Grassland	Frisby 3036; Goyder 8121	
	Combretum psidioides Welw. subsp. psidioides	Grassland	Frisby 3053; Goyder 8345	
	*Combretum* sp. not matched 1	Woodland	Goyder 8307	
	*Combretum* sp. not matched 2	Grassland	Goyder 8346	
	*Pteleopsisanisoptera* (Welw. ex M.A.Lawson) Engl. & Diels	Woodland	Goyder 8418	
	*Terminalia brachystemma* Welw. ex Hiern	Woodland	Frisby 3011; Goyder 8378	
** Compositae **	*Anisopappuschinensis* Hook. & Arn.	Grassland	Goyder 8908	
	*Bidenscrocea* Welw. ex O.Hoffm.	Woodland	Goyder 8253	
	*Blumeaaxillaris* (Lam.) DC.	Grassland	Barker et al. 134	
	*Crassocephalum* sp. not matched	Woodland	Goyder 8305	
	*Dicomaschinzii* O.Hoffm.	Grassland	Barker et al. 85	
	*Emiliabaumii* (O.Hoffm.) S.Moore	Woodland	Baum 707; Goyder 8252; Goyder 8910	Moxico
	*Erlangiamisera* (Oliv. & Hiern) S.Moore	Woodland	Barker et al. 125	
	*Hypericophyllumgossweileri* S.Moore	Grassland	Goyder 8948	Angola
	*Mikaniasagittifera* B.L.Robb.	Grassland	Barker et al. 104; Baum 679	
	*Nidorellaresedifolia* DC.	Grassland	Barker et al. 126	
	*Pasaccardoabaumii* O.Hoffm.	Grassland	Frisby 3013; Goyder 8111	
	*Pleiotaxislinearifolia* O. Hoffm.	Grassland Woodland	Barker et al. 69; Barker et al. 120	
	*Pleiotaxisrugosa* O.Hoffm.	Woodland	Barker et al. 75	
	*Pleiotaxissubscaposa* C.Jeffrey	Grassland	Goyder 8279; Goyder 8456	Moxico
	*Pseudognaphaliumluteoalbum* (L.) Hilliard & B.L.Burtt	Grassland	Barker et al. 70; Frisby 3019	
	*Seneciostrictifolius* Hiern	Wetland	Barker et al. 110; Barker et al. 127; Goyder 8915	
	*Vernonia* sp. nov.	Wetland	Goyder 8357	
	*Vernonia* sp.	Grassland	Goyder 8459	
	Vernonia gerberiformis Oliv. & Hiern subsp. gerberiformis var. gerberiformis	Grassland	Goyder 8109	
	*Vernoniaornata* S.Moore	Wetland	Frisby 3091	
	Vernonia poskeana Vatke & Hildebr. subsp. poskeana	Woodland	Barker et al. 84	
	*Vernoniasubplumosa* O.Hoffm.	Woodland	Baum 703; Goyder 8286	Moxico
	*Vernoniaturbinella* S.Moore	Woodland	Goyder 8017	
** Convolvulaceae **	*Ipomoeawelwitschii* Vatke ex Hallier f.	Grassland	Goyder & Maiato 8828	Moxico
** Cucurbitaceae **	*Acanthosicyosnaudinianus* (Sond.) C. Jeffrey	Ruderal; Grassland	Barker et al. 119; Goyder 8086	
** Dilleniaceae **	*Tetracerapoggei* Gilg	Woodland	Goyder 8021; Goyder 8214	Bié; Moxico
** Dipterocarpaceae **	*Monotesdasyanthus* Gilg	Woodland	Goyder 8039; sight record 34	
	*Monotesglaber* Sprague	Woodland	Goyder 8014; Goyder 8122; sight record 20; sight record 33	
	*Monotesgossweileri* De Wild.	Grassland	Goyder 8338; Goyder 8951	
** Droseraceae **	*Droseraaffinis* Welw. ex Oliv.	Wetland	Baum 687; Goyder 8260; Goyder 8356	Moxico
	*Droseraburkeana* Planch.	Wetland	Goyder & Maiato 8794	
	*Droseramadagascariensis* DC.	Wetland	Frisby 4011; Goyder 8003; Goyder 8006; Goyder 8372; sight record 40; Goyder & Maiato 8786	
** Ebenaceae **	*Diospyrosbatocana* Hiern	Woodland	Barker et al. 142; Goyder 8029	
	*Diospyroschamaethamnus* Dinter ex Mildbr.	Grassland	Goyder 8901	
	DiospyrospseudomespilusMildbr.subsp.brevicalyx F.White	Woodland	Goyder 8032; sight record 32	
	*Diospyrosvirgata* (Gürke) Brenan	Woodland	Goyder 8015	
** Ericaceae **	Erica benguelensis (Welw. ex Engl.) E.G.H.Oliv. var. benguelensis	Grassland	Goyder 8352	
** Euphorbiaceae **	*Acalypha* sp. not matched	Grassland	Goyder & Maiato 8802; Goyder & Maiato 8814	
	*Maprouneaafricana* Müll.Arg. pyrophytic form	Grassland	Goyder 8312	
	*Sclerocrotonoblongifolius* (Müll.Arg.) Kruit & Roebers	Grassland	Goyder 8314; Goyder & Maiato 8844	
** Gentianaceae **	*Faroasalutaris* Welw.	Wetland; Grassland	Barker et al. 53; Frisby 4000; Goyder 8216	
	*Neurothecacongolana* De Wild. & T.Durand	Wetland	Goyder 8234; Goyder 8354	Moxico
	*Pycnosphaerabuchananii* (Baker) N.E.Br.	Wetland	Goyder 8462	
	*Schinziellatetragona* (Schinz) Gilg	Wetland	Goyder 8333; Goyder 8355	
** Gisekiaceae **	*Gisekiaafricana* (Lour.) Kuntze	Grassland	Barker et al. 124; Goyder 8233; Goyder 8949	Moxico
** Hypericaceae **	*Hypericumoligandrum* Milne-Redh.	Wetland	Frisby 4026	
	*Psorospermumbaumii* Engl.	Woodland	Frisby 4003; Goyder 8221	Bié
** Ixonanthaceae **	*Ochthocosmus lemaireanus T.Durand & H.Durand*	Woodland	Barker et al. 48; Barker et al. 74; Baum 712; Goyder 8095; Goyder 8311; Goyder 8313; sight record 27	Moxico
** Lamiaceae **	*Alvesiarosmarinifolia* Welw.	Woodland	Barker et al. 45; Baum 676; Goyder 8036	
	*Clerodendrumbaumii* Gürke	Grassland	Baum 661; Goyder 8125; Goyder 8367	
	*Clerodendrumbuchneri* Gürke	Grassland	Goyder 8262	
	*Clerodendrumformicarum* Gürke	Grassland	Goyder & Maiato 8798	
	*Endostemon* sp. nov.	Grassland	Goyder & Maiato 8762	
	*Haumaniastrumkatangense* (S.Moore) J.Duvign. & Plancke	Grassland	Goyder 8903	
	Haumaniastrumprealtum(Briq.)J.Duvign. & Planckevar.succisifolium (Baker) A.J.Paton	Grassland	Goyder 8341; Goyder 8454	Moxico
	*Haumaniastrumsericeum* (Briq.) A.J.Paton	Grassland	Barker et al. 87; Goyder 8440	
	*Kalahariauncinata* (Schinz) Moldenke	Grassland	Goyder & Maiato 8782	
	Leonotis nepetifolia (L.) R.Br. var. nepetifolia	Ruderal	Baum 822	
	Ocimum obovatum E.Mey. ex Benth. var. obovatum	Grassland	Goyder & Maiato 8787	
	*Plectranthusbetonicifolius* Baker	Wetland	Goyder 8463	Moxico
	*Plectranthusgracillimus* (T.C.E.Fr.) Hutch. & Dandy	Grassland	Goyder 8902	
	*Plectranthusguerkei* Briq.	Grassland	Barker et al. 86	
	*Plectranthusmirabilis* (Briq.) Launert	Wetland	Barker et al. 140; Baum 794; Goyder 8007; Goyder 8928	
	*Pycnostachysgracilis* R.D.Good	Woodland	Goyder 8441	
	*Tinneaeriocalyx* Welw.	Grassland	Goyder 8250	
	*Tinneafusco-luteola* Gürke	Grassland	Baum 695	
	*Tinneabenguellensis* Gürke	Grassland	Baum 697; Goyder 8458	Moxico
	VitexmadiensisOliv.subsp.milanjiensis (Britten) F.White	Woodland	Frisby 3023; Frisby 3046; Goyder 8044; Goyder 8416; Goyder 8428	
** Lecythidaceae **	*Napoleonaeagossweileri* Baker f.	Grassland	Goyder 8107; Goyder & Maiato 8812	Moxico
** Leguminosae **	*Aeschynomenedimidiata* Welw. ex Baker	Woodland	Goyder 8392	Moxico
	*Aeschynomeneglabrescens* Welw. ex Baker	Wetland	Goyder & Maiato 8784	
	*Albiziaadianthifolia* (Schumach.) W.Wight	Woodland	Goyder 8212	
	Baphia massaiensis Taub. subsp. obovata (Schinz) Brummitt var. obovata	Woodland	Frisby 3024; Goyder 8092; Goyder 8449; sight record 7; sight record 31; Goyder & Maiato 8780	
	*Baphia* sp. nov.	Grassland	Goyder & Maiato 8772	
	*Bauhiniamendoncae* Torre & Hillc.	Woodland	Barker et al. 76; Goyder 8030; Goyder 8391	
	BauhiniapetersianaBollesubsp.macrantha (Oliv.) Brummitt & J.H. Ross	Woodland	Frisby 4017	
	*Bobgunniamadagascariensis* (Desv.) J.H. Kirkbr. & Wiersema	Woodland	Goyder 8031; Goyder 8384; Goyder 8429; Goyder 8450	
	*Brachystegiabakeriana* Hutch. & Burtt Davy	Woodland	Barker et al. 100; Frisby 3014; Goyder 8020; Goyder 8090; Goyder 8116; Goyder 8386; Goyder 8430; Goyder 8432; Goyder 8448; sight record 10	
	*Brachystegialongifolia* Benth.	Grassland; Woodland	Goyder 8011; Goyder 8328; Goyder 8921	
	*Brachystegiaspiciformis* Benth.	Woodland	Goyder 8038	
	*Burkeaafricana* Hook.	Grassland	Goyder 8379; sight record 37; sight record 43	
	*Chamaecristamimosoides* (L.) Greene sens. lat.	Woodland	Barker et al. 83	
	*Clitoriakaessneri* Harms – depauperate form	Grassland	Goyder & Maiato 8758	
	*Copaiferabaumiana* Harms	Grassland; Woodland	Goyder 8018; Goyder 8113; Goyder 8224; Goyder 8388; sight record 3; Goyder & Maiato 8847; Goyder 8919	
	*Crotalariaabscondita* Welw. ex Baker	Grassland	Goyder 8465	Moxico
	*Crotalariaangulicaulis* Harms	Grassland	Goyder 8452	Moxico
	*Crotalariaannua* Milne-Redh.	Grassland	Goyder 8900	
	*Crotalariakambolensis* Baker f.	Woodland	Goyder 8424	
	*Crotalarialeptoclada* Harms	Grassland	Baum 829	
	*Crotalariamendoncae* Torre	Woodland	Goyder 8016; Goyder 8103; sight record 26	Cuando Cubango
	*Crotalariastenoptera* Welw. ex Baker	Grassland; Wetland; Woodland	Barker et al. 146; Baum 677; Goyder 8093; Goyder 8257	
	*Crotalariayoungii* Baker f.	Grassland; Woodland	Goyder 8218	Bié
	Crotalariacf.youngii Baker f.	Grassland; Woodland	Barker et al. 144; Goyder 8944	
	CryptosepalumexfoliatumDe Wild.subsp.pseudotaxus (Baker f.) P.A.Duvign. & Brenan	Woodland	Goyder 8022; Goyder 8323; Goyder 8446; sight record 4; sight record 12; sight record 24	
	*Cryptosepalummimosoides* Welw. ex Oliv.	Grassland	Goyder 8337; Goyder & Maiato 8751	Moxico
	Desmodiumbarbatum(L.)Benth.var.dimorphum (Welw. ex Baker) B.G.Schub.	Grassland	Baum 685; Goyder 8502	
	*Dialiumenglerianum* Henriq.	Woodland	Goyder & Maiato 8805	
	Entada arenaria Schinz subsp. arenaria	Grassland; Woodland	Goyder 8390; Goyder & Maiato 8836	Moxico
	*Erythrophleumafricanum* (Welw. ex Benth.) Harms	Woodland	Goyder 8010; Goyder 8380; Goyder 8389; sight record 29; Goyder 8922	
	*Erythrinabaumii* Harms	Grassland	Frisby 4034; Goyder & Maiato 8767	
	*Guibourtiacoleosperma* (Benth.) J.Léonard	Woodland	Goyder 8035; Goyder 8377; sight record 2; sight record 13; sight record 23; sight record 30; sight record 35	
	*Indigoferabaumiana* Harms	Grassland	Baum 819; Goyder & Maiato 8818	
	*Indigoferasutherlandioides* Baker	Woodland	Goyder 8046; Goyder 8955	
	*Kotschyastrobilantha* (Welw. ex Baker) Dewit & P.A.Duvign.	Grassland	Barker et al. 56; Goyder 8091; Goyder 8943	
	*Julbernardiapaniculata* (Benth.) Troupin	Woodland	Goyder 8012; Goyder 8089; Goyder 8123; Goyder 8308; Goyder 8443; sight record 11; sight record 19	
	*Macrotylomarupestre* (Welw. ex Baker) Verdc.	Woodland	Goyder 8247	Moxico
	*Pterocarpusangolensis* DC.	Woodland	Barker et al. 52B; Goyder 8009; Goyder 8382; sight record 6; sight record 22; sight record 28	
	*Rhynchosiaprocurrens* (Hiern) K.Schum.	Woodland	Barker et al. 77	
	Sphenostyliserecta(Baker f.)Hutch. ex Baker f.subsp.obtusifolia (Harms) Potter & Doyle	Woodland	Goyder 8248; Goyder 8950	Moxico
** Lentibulariaceae **	*Genliseaangolensis* R.D.Good	Wetland	Frisby 3073; Goyder 8120; Goyder 8315; Goyder 8371	Moxico
	*Utriculariagibba* L.	Wetland	Barker et al. 44a; Goyder 8098	
	*Utriculariaspiralis* Sm.	Wetland	Frisby 3094; Goyder 8114	
	*Utriculariasubulata* L.	Wetland	Baum 691; Goyder 8370	
	*Utriculariastellaris* L.f.	Wetland	Frisby 3088	
** Limeaceae **	*Limeumfenestratum* (Fenzl) Heimerl	Grassland	Barker et al. 80; Baum 688	
** Linderniaceae **	*Crepidorhopalon* ?sp. nov.	Grassland	Goyder 8917	
** Loranthaceae **	*Englerinagabonensis* (Engl.) Balle	Woodland	Goyder 8413	
	*Tapinanthusdependens* (Engl.) Danser	Woodland	Barker et al. 137	
** Lythraceae **	*Rotalamyriophylloides* Welw. ex Hiern	Wetland	Barker et al. 68	
** Malvaceae **	*Grewiafalcistipula* K.Schum.	Woodland	Frisby 3022	
	*Grewia* sp.	Ruderal	Goyder & Maiato 8819	
	*Triumfettadekindtiana* Engl.	Woodland	Barker et al. 133	
** Melastomataceae **	*Antherotomadebilis* (Sond.) Jacq.-Fél.	Wetland	Barker et al. 47; Frisby 4031; Goyder 8094	
	*Dissotisbrazzae* Cogn.	Grassland	Goyder 8927	
	Dissotis rhinanthifolia (Brenan) A.Fern. & R.Fern. var. rhinanthifolia	Wetland	Goyder & Maiato 8823	
	*Dissotiswelwitschii* Cogn.	Wetland	Goyder 8240	Moxico
	*Memecylonhuillense* A.Fern. & R.Fern.	Woodland	Goyder 8399	Moxico
** Meliaceae **	*Trichiliaquadrivalvis* C.DC.	Woodland	Frisby 3070; Goyder & Maiato 8839	Moxico
** Menyanthaceae **	*Nymphoidesforbesiana* (Griseb.) Kuntze	Wetland	Goyder & Maiato 8824	Moxico
	Nymphoidesindica(L.)Kuntzesubsp.occidentalis A.Raynal	Wetland	Barker et al. 113	
** Moraceae **	*Ficuspygmaea* Welw. ex Hiern	Wetland	Barker et al. 141	
	*Ficusverruculosa* Warb.	Wetland	Goyder 8320	
** Myricaceae **	*Morellaserrata* (Lam.) Killick	Wetland	Goyder 8914	
** Myrtaceae **	Syzygium cordatum Hochst. ex Krauss subsp. cordatum	Wetland	Barker et al. 145; Goyder 8319	
	Syzygiumguineense(Willd.)DC.subsp.huillense (Hiern) F.White	Grassland	Barker et al. 67; Frisby 3045; Goyder 8339; Goyder & Maiato 8835	
	*Syzygiumowariense* (P.Beauv.) Benth.	Humid Forest	Goyder 8326	Moxico
** Ochnaceae **	*Brackenridgeaarenaria* (De Wild. & T.Durand) N.Robson	Grassland	Frisby 3015; Frisby 3016; Frisby 3060; Goyder & Maiato 8781; Goyder & Maiato 8804	
	*Ochnakatangensis* De Wild.	Grassland	Goyder & Maiato 8754A	
	*Ochnamanikensis* De Wild.	Grassland	Frisby 3031; Goyder 8108; Goyder 8309	Moxico
	*Ochnapulchra* Hook.	Woodland	Goyder 8013; Goyder 8383; sight record 21	Moxico
	*Ochnapygmaea* Hiern	Grassland	Frisby 3059; Goyder & Maiato 8754B	Moxico
** Olacaceae **	*Olaxgossweileri* Exell & Mendonça	Woodland	Goyder & Maiato 8846	Moxico
** Oleaceae **	OleacapensisL.subsp.macrocarpa (C.H.Wright) I.Verd.	Woodland	Goyder 8437	Moxico
** Onagraceae **	*Ludwigiaoctovalvis* (Jacq.) P.H.Raven	Wetland	Barker et al. 138	
** Orobanchaceae **	*Buchneraprorepens* Engl. & Gilg	Grassland	Goyder 8349; Goyder 8451	Moxico
	*Buchneraattenuata* Skan	Wetland	Frisby 3086; Frisby 4021	
	*Buchnera* sp. not matched at K	Grassland	Goyder 8276	
	*Buchnerawelwitschii* Engl.	Grassland	Barker et al. 93	
	Cycnium tubulosum (L.f.) Engl. subsp. tubulosum	Grassland	Frisby 4019	
	*Gerardiinaangolensis* Engl.	Wetland	Goyder 8101	
	*Gerardiinaangolensis* Engl. – unusual form with branched inflorescence	Wetland	Goyder 8293	
	*Melasmacalycinum* (Hiern) Hemsl.	Wetland	Frisby 4018; Goyder & Maiato 8760	
	*Micrargeriellaaphylla* R.E.Fr.	Wetland	Goyder & Maiato 8783	Moxico
	*Sopubiasimplex* (Hochst.) Hochst.	Wetland	Frisby 3083; Frisby 4024; Goyder & Maiato 8821	Moxico
	*Strigaangolensis* K.I.Mohamed & Musselman	Wetland	Goyder 8336	
	*Strigabilabiata* (Thunb.) Kuntze	Wetland	Frisby 4028; Goyder & Maiato 8795	Moxico
** Passifloraceae **	Basananthebaumii(Harms)W.J. de Wildevar.caerulescens (A.Fern. & R.Fern.) W.J. de Wilde	Grassland	Goyder & Maiato 8826	
** Passifloraceae **	*Paropsiabrazzaeana* Baill.	Woodland	Barker et al. 101; Goyder 8024; sight record 25; Goyder 8920	
** Pedaliaceae **	*Sesamumcalycinum* Welw.	Grassland	Frisby 4022	
** Peraceae **	*Clutiabenguelensis* Müll.Arg.	Grassland	Goyder 8455	Moxico
** Phrymaceae **	*Mimulusgracilis* R.Br.	Wetland	Barker et al. 135	
** Phyllanthaceae **	*Brideliaduvigneaudii* J.Léonard	Woodland	Goyder 8423	Moxico
	*Hymenocardiaacida* Tul.	Woodland	Goyder 8231	
	*Uapacanitida* Müll.Arg.	Woodland	Goyder 8047; Goyder 8427	
	*Uapacanitida* Müll.Arg. – pyrophytic form	Grassland Woodland	Goyder 8217	
** Picodendraceae **	*Oldfieldiadactylophylla* (Welw. ex Oliv.) J.Léonard	Grassland	Goyder 8267; Goyder 8421	
** Plantaginaceae **	*Limnophyllaceratophylloides* (Hiern) Skan	Wetland	Goyder 8318	Moxico
** Polygalaceae **	*Polygalaafricana* Chodat	Wetland	Frisby 4027	
	*Polygalaarenicola* Gürke	Woodland	Barker et al. 123; Goyder 8229	
	*Polygaladewevrei* Exell	Wetland	Goyder 8361 (blue fls); Goyder 8362 (white fls); Goyder & Maiato 8849; Goyder 8926	Bié
	*Polygalagomesiana* Welw. ex Oliv.	Wetland	Goyder 8374	Cuando Cubango
	*Polygalakalaxariensis* Schinz	Grassland	Barker et al. 96	
	*Polygalamendoncae* E.M.A.Petit	Woodland	Goyder 8037; Goyder 8417	
	*Polygalanambalensis* Gürke	Grassland	Goyder 8453	Moxico
	*Polygalanematophylla* Exell	Grassland	Goyder 8366	Moxico
	*Polygalapaludicola* Gürke	Wetland	Barker et al. 92; Frisby 3040; Frisby 3085; Goyder 8119; Goyder 8236	
	*Polygalapoggei* Gürke	Grassland	Goyder 8278	Moxico
	*Polygalarivularis* Gürke	Grassland	Barker et al. 90	
	*Polygalarobusta* Gürke	Grassland	Baum 704; Frisby 3047; Goyder 8085; Goyder 8280; Goyder 8303; Goyder 8411	Moxico
	*Polygalaspicata* Chodat	Wetland	Frisby 3084; Goyder 8235; Goyder 8363	
	PolygalawelwitschiiChodatsubsp.pygmaea (Gürke) Paiva	Grassland	Goyder 8350; Goyder 8916	Moxico
	*Securidacalongipedunculata* Fresen.	Woodland	sight record 44	
** Polygonaceae **	*Oxygonumannuum* S.Ortíz & Paiva	Grassland	Goyder 8348	Moxico
	*Oxygonumfruticosum* Dammer ex Milne-Redh.	Woodland	Goyder 8008; Goyder 8105; Goyder 8954	
	*Oxygonumpachybasis* Milne-Redh.	Grassland	Frisby 3090; Goyder & Maiato 8799	Moxico
** Proteaceae **	*Faureadelevoyi* De Wild.	Wetland; Woodland	Goyder 8398	
	*Faureasaligna* Harv.	Woodland	Barker et al. 102	
	Protea angolensis Welw. var. angolensis	Grassland	Goyder 8410	Moxico
	Protea baumii Engl. & Gilg subsp. baumii	Grassland	Barker et al. 106	
	Protea petiolaris (Hiern) Baker & C.H.Wright subsp. petiolaris	Grassland	Goyder 8412	Moxico
	ProteapoggeiEngl.subsp.haemantha Chisumpa & Brummitt	Woodland	Baum 709; Goyder 8215; sight record 14; Goyder 8956	Bié; Moxico
	*Proteawelwitschii* Engl.	Grassland	Goyder 8117; Goyder 8353; Goyder 8397; Goyder 8460	Moxico
** Ranunculaceae **	*Clematisvillosa* DC.	Grassland	Goyder 8912	
** Rosaceae **	CliffortianitidulaR.E.Fr. & T.C.E.Fr.var.angolensis (Weim.) Brenan	Grassland	Barker et al. 103; Baum 650; Goyder 8395; Goyder & Maiato 8855; Goyder 8932	
** Rubiaceae **	*Ancylanthosrubiginosus* Desf.	Grassland Woodland	Frisby 3056; Goyder 8115; Goyder & Maiato 8775; Goyder & Maiato 8848	
	*Bertiera* sp.	Humid Forest	Goyder 8325	
	*Diodiaflavescens* Hiern	Grassland	Barker et al. 99	
	*Fadogiacienkowski* Schweinf.	Grassland	Frisby 3018; Goyder 8501	
	*Fadogiafuchsioides* Oliv.	Grassland	Goyder 8340	
	*Fadogiagossweileri* Robyns	Woodland	Frisby 4032	
	FadogiatomentosaDe Wild.var.flaviflora (Robyns) Verdc.	Woodland	Goyder 8246	
	*Gangueliagossweileri* (S.Moore) Robbr.	Grassland	Goyder & Maiato 8815	
	*Gardeniaimperialis* K.Schum.	Wetland	Goyder 8321; Goyder 8394; sight record 39	
	Gardenia resiniflua Hiern subsp. resiniflua	Woodland	Barker et al. 143	
	GardeniaresinifluaHiernsubsp.resiniflua – suffrutescent form	Woodland	Barker et al. 51; Frisby 4007	
	*Leptactinabenguelensis* (Benth. & Hook.f.)R.D.Good	Woodland	Frisby 4029; Goyder & Maiato 8842	
	*Morindaangolensis* (R.D.Good) F.White	Grassland	Goyder & Maiato 8756; Goyder & Maiato 8851	Moxico
	*Pavettanitidula* Hiern	Woodland	Goyder & Maiato 8840	
	*Pavetta* sp. 1	Woodland	Goyder 8242	
	*Pavetta* sp. 2	Woodland	Goyder 8249	
	*Pavetta* sp. 3	Grassland	Goyder 8301	
	*Psychotria* sp.	Humid Forest	Goyder 8324	
	*Psydraxgilletii* (De Wild.) Bridson	Woodland	Goyder 8434	
	*Psydrax* sp.	Woodland	Goyder 8433	
	*Pygmaeothamnuszeyheri* (Sond.) Robyns	Grassland	Goyder & Maiato 8808; Goyder & Maiato 8811	
	Rothmannia engleriana (K.Schum.) Keay var. engleriana	Woodland	Goyder 8420	
	*Rytigyniaorbicularis* (K.Schum.) Robyns	Woodland	Goyder 8227	
	*Tricalysiaangolensis* A.Rich. ex DC.	Woodland	Barker et al. 73	
	*Tricalysia* sp.	Woodland	Goyder 8435	
	*Vangueria* sp. not matched at K	Woodland	Goyder 8265	
	Vangueriopsiscf.lanciflora (Hiern) Robyns	Woodland	Goyder 8422	
** Rutaceae **	*Zanthoxylumgilletii* (De Wild.) P.G.Waterman	Humid Forest	Goyder 8327	Moxico
** Santalaceae **	*Thesiumatrum* A.W.Hill	Grassland	Goyder 8342; Goyder & Maiato 8813	Moxico
	*Thesiumsubaphyllum* Engl.	Grassland	Barker et al. 91; Goyder 8347; Goyder 8937	
** Sapotaceae **	*Chrysophyllumbangweolense* R.E.Fr.	Woodland	Goyder & Maiato 8841	
	*Englerophytummagalismontanum* (Sond.) T.D.Penn.	Woodland	Goyder 8033; Goyder 8387; Goyder 8447; sight record 5	
	*Englerophytummagalismontanum* (Sond.) T.D.Penn. – pyrophytic form	Grassland Woodland	Goyder & Maiato 8854	
** Simaroubaceae **	*Hannoachlorantha* Engl. & Gilg	Woodland	Barker et al. 66; Barker et al. 129; Baum 674; Goyder 8946	Moxico
** Thymelaeaceae **	*Craterosiphonquarrei* Staner	Woodland	Goyder 8219; Goyder & Maiato 8845	Moxico
	Gnidia gossweileri (S.Moore) B.Peterson subsp. gossweileri	Wetland; Grassland	Barker et al. 88	
	*Gnidiakraussiana* Meisn.	Grassland	Goyder 8110; Goyder & Maiato 8817	
** Umbelliferae **	*Afrocarumimbricatum* (Schinz) Rauschert	Wetland	Goyder 8957	
	*Pseudoselinumangolense* (C.Norman) C.Norman	Grassland; Woodland	Goyder 8045; Goyder 8251; Goyder 8953	Bié; Moxico
